# Comparison of multichannel signal deconvolution algorithms in airborne LiDAR bathymetry based on wavelet transform

**DOI:** 10.1038/s41598-021-96551-w

**Published:** 2021-08-20

**Authors:** Yue Song, Houpu Li, Guojun Zhai, Yan He, Shaofeng Bian, Wei Zhou

**Affiliations:** 1 Department of Navigation Engineering, Naval University of Engineering, Wuhan, 430033 China; 2 Naval Institute of Marine Environment, Tianjin, 300061 China; 3 Key Laboratory of Space Laser Communication and Detection Technology, Shanghai Institute of Optics and Fine Mechanics, Chinese Academy of Sciences, Shanghai, 201800 China; 4 Guangxi Key Laboratory of Spatial Information and Geomatics, Guilin, 541004 China

**Keywords:** Applied optics, Lasers, LEDs and light sources, Optical techniques

## Abstract

Airborne LiDAR bathymetry offers low cost and high mobility, making it an ideal option for shallow-water measurements. However, due to differences in the measurement environment and the laser emission channel, the received waveform is difficult to extract using a single algorithm. The choice of a suitable waveform processing method is thus of extreme importance to guarantee the accuracy of the bathymetric retrieval. In this study, we use a wavelet-denoising method to denoise the received waveform and subsequently test four algorithms for denoised-waveform processing, namely, the Richardson–Lucy deconvolution (RLD), blind deconvolution (BD), Wiener filter deconvolution (WFD), and constrained least-squares filter deconvolution (RFD). The simulation and measured multichannel databases are used to evaluate the algorithms, with focus on improving their performance after data-denoising and their capability of extracting water depth. Results show that applying wavelet denoising before deconvolution improves the extraction accuracy. The four algorithms perform better for the shallow-water orthogonal polarization channel (PMT2) than for the shallow horizontal row polarization channel (PMT1). Of the four algorithms, RLD provides the best signal-detection rate, and RFD is the most robust; BD has low computational efficiency, and WFD performs poorly in deep water (< 25 m).

## Introduction

Airborne LiDAR bathymetry (ALB) is an active remote-sensing technology that plays an important role in shallow-water topographic surveys and measurements. It boasts advantages of low operating cost, strong maneuverability, and high measurement accuracy, and is thus widely applied to update coastal topographic maps, for coastal construction, to monitor shallow waters, and in other fields^1–3^. ALB uses a strong penetrating green laser (532 nm) to scan waters less than 50 m deep. ALB emits blue and green laser beams and receives signals reflected off various targets. The characteristic information is extracted from the difference in signal strength, and the technique is applied to measuring water depth and underwater topography, as well as providing low-quality classifications of the seabed^4–6^. A laser beam transmitted over a few hundred meters passes through the air and water to the seabed and is reflected back to the receiver. Along this path, a variety of noises perturb the signal, including noise internal to the receiver, atmospheric refraction, backscattering from water bodies, and diffuse reflection from the seabed. ALB is more complicated than terrestrial LiDAR measurements. Therefore, to process full-waveform ALB data, a crucial step is to improve the signal-to-noise ratio (SNR) of the data and accurately extract the echo signal^7^.

In early airborne laser scanning (ALS) systems, only the transmitted signal and echo position signal were accepted; the complete echo signal was not retained to avoid storing redundant data. Pe’Eri proposed the use of two waveforms for measurements in shallow waters. Near-infrared laser pulses are strongly reflected at the water surface, whereas a green laser beam penetrates the water more easily, after which it is reflected from the seabed^8^. Wong proposed using low-pass filters to remove noise from the signal and thereby extract the signal reflected from the seabed^9,10^. However, retaining only the transmitted and echo position signals may reduce target resolution, ranging accuracy, etc. Simultaneously, copious information about the water body (such as the turbidity) cannot be extracted from this measurement. The study of Mallet shows the importance of using full-wave laser data to measure seafloor topography. He proposes that full-wave laser data are more conducive to extracting the target position and for research on backscattering from water bodies^3,11^. Characterization of full-wave laser data is thus important to increase our understanding of the laser beam propagation. At present, processing algorithms for full-wave green laser data can be sorted into the following three categories:

- Target-detection method: Depending on the original waveform, the target-detection method extracts the reflected laser energy time point by identifying the mutation point of the continuous signal reflection energy. Common target-detection methods include peak detection (PD), first-order derivative (FD), and the average square function (ASDF)^12–14^. The algorithm is fast; however, it is susceptible to gross errors and noise. It is generally used to extract the initial value and extract the reflection time point after data preprocessing.
- Waveform decomposition method: The waveform decomposition method treats the laser waveform as a superposition of multiple mathematical functions and uses a combination of multiple mathematical functions to fit the waveform, thereby restoring the true original signal. Commonly used fitting functions include Gaussians, logarithmic normal distribution functions, and quadrilateral functions^15–17^. Gaussian functions are widely used for fitting; for example, Gaussian fitting based on the nonlinear least-squares method^18^, Kai Guo based on Gaussian fitting^19^, and Gaussian half-wave width to decompose the waveform and extract the effective part of the data to suppress noise, which somewhat improves the problem of Gaussian overfitting. Wang^14^ compared quadrilateral fitting with mean variance fitting; however, the waveform fitting algorithm has difficulty in solving the problem of signal overlap, such as the overlap of surface and bottom echoes. The backscattering of the water body is an asymmetric waveform, making it difficult to fit the Gaussian function.
- Deconvolution algorithm: The deconvolution algorithm regards the received waveform as the convolution of the laser emission pulse and the target cross-section. Commonly used deconvolution algorithms include the Wiener filter deconvolution^20^, Richardson–Lucy deconvolution (RLD)^21^, blind deconvolution (BD)^14^, and B-spline deconvolution^22^. However, the deconvolution algorithm is more affected by noise, which leads to misjudgment of reflected echoes, and the data processing results are easily affected by ringing.

The three types of data processing methods for green lasers show that target detection is relatively simple and does not depend on the laser propagation process; however, their accuracy is poor. The waveform decomposition method provides an approximate fit to the full-waveform data, and most waveform fitting functions are symmetrical functions. The waveform decomposition method does not consider backscattering of the water body, which causes unsatisfactory fitting. In contrast, the deconvolution algorithm starts from the laser propagation and waveform formation process and uses the inverse transformation of the waveform formation to restore a more realistic target cross-section. If noise can be removed, the deconvolution algorithm is an ideal method for laser echo processing. At present, the wavelet theory is widely employed in digital signal processing^23^.

This study first establishes a water-depth radar model (Wa-LiD) that is close/to reality according to the airborne laser propagation and formation process and then applies wavelet denoising to the simulation results to find the best denoising parameters. Subsequently, the denoised data are deconvoluted, the advantages and disadvantages of various algorithms are compared, and the influence of the parameters on the echo received by the laser is analyzed. Finally, the results of applying the algorithm to the simulation and measured data are comprehensively evaluated to determine the optimal deconvolution method. This lays the foundation for extracting water depth by airborne laser.

## Materials and methods

### Simulated and acquisition dataset

#### Simulated dataset

The Wa-LiD model is simulated by applying the reflection and refraction of different wavelengths in a complex water environment to form a reflected wave. The result of the simulation is close to the actual ALB waveform and can amplify the influence of various factors on the waveform, which facilitates the comparison of various parameters in waveform denoising and deconvolution. During the actual flight measurement, as noise-free waveform data is impossible to obtain, the accuracy of the algorithm is often evaluated using statistically relative truth values, and the simulation data can generate completely-noise-free simulation truth values, which promotes the accuracy of the algorithm. The evaluation, therefore, is particularly important to obtain laser simulation results that are closer to reality. The wavelength range of the Wa-LiD model^24^ is 300–1500 nm. The laser used by the ALB system produces long-wave 1064 nm infrared light and 532 nm blue-green light. Infrared light cannot penetrate the water body and is reflected from its surface. Blue-green light penetrates the water body and reflects from the bottom. Therefore, the model uses 532 nm green light, which has strong penetrability. The echo waveform of the ALB system can be expressed as the superposition of multiple echoes:

1 $${p}_{r}(\mathrm{t})={p}_{s}\left(\mathrm{t}\right)+{p}_{c}\left(\mathrm{t}\right)+{p}_{b}\left(\mathrm{t}\right)+{p}_{N}\left(\mathrm{t}\right),$$

where ${p}_{s}\left(\mathrm{t}\right)$ is reflection from the water surface, ${p}_{c}\left(\mathrm{t}\right)$ is reflection from the water column scattering, ${p}_{b}\left(\mathrm{t}\right)$ is reflection from the bottom, and ${p}_{N}\left(\mathrm{t}\right)$ is the ALB system noise. The laser radar emission pulse can be expressed by a Gaussian function^20^:

2 $${w}_{t}\left({t}_{x}\right)=\frac{2}{{T}_{0}}\sqrt{\frac{\mathrm{ln}2}{\pi }}\mathrm{exp}\left(-4ln2\frac{{(t-{t}_{x})}^{2}}{{T}_{0}^{2}}\right),$$

where ${T}_{0}$ is the full width at half maximum of the transmitted pulse, and ${t}_{x}$ is the round-trip time required for the transmitted pulse to reach the target and return.

1. The water-surface reflection model
  After the laser pulse is emitted from the transmitter, it passes through the air and reflects from the water surface. The reflected energy received by the ALB detector can be expressed as the convolution of the transmitted pulse and the water surface echo energy ${p}_{s}$:
  3 $${p}_{s}\left(t\right)=w\left({t}_{s}\right) \otimes {P}_{s},$$
  where \otimes indicates the convolution, and ${p}_{s}$ is
  4 $${p}_{s}=\frac{{P}_{e}{T}_{\mathrm{atm}}^{2}{A}_{R}{\eta }_{e}{\eta }_{R}{L}_{S}{\mathrm{cos}}^{2}({i}_{\mathrm{laser}})}{\pi {H}^{2}},$$
  where ${P}_{e}=\frac{{E}_{0}}{{T}_{0}}$ is the laser emission power, ${E}_{0}$ is the laser emission energy, ${T}_{\mathrm{atm}}^{2}$ is the atmospheric double-pass loss factor, ${A}_{R}$ is the receiving area of the receiver, ${\eta }_{e}$ is the optical emission efficiency of the laser transmitter, ${\eta }_{R}$ is the optical receiving efficiency of the receiver, ${i}_{\mathrm{laser}}$ is the incident angle of the laser beam with respect to the normal of the water surface, $H$ is the sensor height, and ${L}_{S}$ is the laser the transmission loss in traveling to the water surface.
  ${L}_{S}$ is expressed as
  5 $${L}_{s}=\frac{{k}_{d}}{\pi }+\frac{{k}_{s}{e}^{{(-\mathrm{tan}{i}_{\mathrm{laser}}/r)}^{2}}{\alpha }_{\mathrm{BRDF}}{F}_{r}}{\pi {r}^{2}{\mathrm{cos}}^{6}({i}_{\mathrm{laser}})},$$
  where ${k}_{d}$ is the diffuse reflection coefficient of the water surface, and ${k}_{s}$ is the specular reflection coefficient. The relationship between ${k}_{d}$ and ${k}_{s}$ is
  6 $${k}_{d}=1-{k}_{s},$$
  where $r$ is the water surface roughness, ${\alpha }_{\mathrm{BRDF}}$ is the geometric attenuation coefficient of the water surface, and ${F}_{r}$ is the function that describes the Fresnel reflection of light from each microfacet.
2. Water reflection model
  The echo reflection signal from depth *D* received by the ALB system receiver can be expressed as the convolution of the transmitted pulse and the instantaneous echo energy at *D*, which is expressed as
  7 $${p}_{c}\left(t\right)=w\left({t}_{c}\right) \otimes {p}_{c}\left(D\right).$$
  The instantaneous echo energy at ${D}_{i}$ can be expressed as
  8 $${p}_{c}\left({D}_{i}\right)=\frac{{P}_{e}{T}_{\mathrm{atm}}^{2}{A}_{R}{\eta }_{e}{\eta }_{R}F{\left(1-{L}_{s}\right)}^{2}\beta (\varnothing )\mathrm{exp}\left(\frac{-2k{D}_{i}}{\mathrm{cos}{r}_{\mathrm{laser}}}\right)}{{\left(\frac{{n}_{w}H+{D}_{i}}{\mathrm{cos}{i}_{\mathrm{laser}}}\right)}^{2}},$$
  where $F$ is the field-angle loss factor, $\beta (\mathrm{\varnothing })$ is the volume scattering function. For LiDAR applications, the only scattering angle of importance is 180°, which could be made more specific to LiDAR by replacing $\beta (\mathrm{\varnothing })$ with $\beta (\uppi )$. ${n}_{w}$ is the water refractive index, *H* is the sensor altitude,${r}_{\mathrm{laser}}$ is the angle of refraction of the laser at the water surface, ${D}_{i}$ is the depth reached by the laser beam in the water, and $k$ is the diffuse attenuation coefficient of the water.
3. Underwater reflection model
  The echo energy at the bottom *Z* received by the ALB system receiver can be expressed as the convolution of the echo at *Z* and the transmitted pulse, with the echo energy at *Z* expressed as
  9 $${p}_{b}\left({D}_{i}\right)=\frac{{P}_{e}{T}_{\mathrm{atm}}^{2}{A}_{R}{\eta }_{e}{\eta }_{R}F{\left(1-{L}_{s}\right)}^{2}{R}_{b}\mathrm{exp}\left(\frac{-2kZ}{\mathrm{cos}{r}_{\mathrm{laser}}}\right)}{\pi {\left(\frac{{n}_{w}H+Z}{\mathrm{cos}{i}_{\mathrm{laser}}}\right)}^{2}},$$
  where ${R}_{b}$ is the bottom reflectance.
4. ALB system noise model

The ALB system noise mainly includes the signal level of solar radiation and the internal noise of the instrument. The signal level due to solar radiation is defined as Gaussian white noise with a mean of zero and a standard deviation of one convolved by an instant echo; ${p}_{ba}$ can be expressed as

10 $${p}_{ba}={I}_{s}{A}_{R}{T}_{atm}^{2}(1-{{\gamma }_{r}}^{2})\frac{\pi {\theta }^{2}}{4}{\Delta }_{\lambda }{\eta }_{R},$$

where ${I}_{s}$ is the intensity of the solar reflection from the water body, and ${\Delta }_{\lambda }$ is the bandwidth of the optical filter of the receiver.

The detector internal noise is defined as a normal distribution with a zero mean and a standard deviation ${\sigma }_{N}(\mathrm{t})$ that varies according to the signal level

11 $${\sigma }_{N}(\mathrm{t})=\frac{\sqrt{2eB(\left({p}_{ba}\left(t\right)+{p}_{s}\left(\mathrm{t}\right)+{p}_{c}\left(\mathrm{t}\right)+{p}_{b}\left(\mathrm{t}\right)\right)G+{I}_{d})}}{{R}_{\lambda }}$$

where $e$ is the electron charge $(1.6\times {10}^{-19}C)$,$B$ is the electrical bandwidth of the detector, is the excess noise factor,${I}_{d}$ is the dark current, and ${R}_{\lambda }$ is the responsivity.

**Figure 1:**
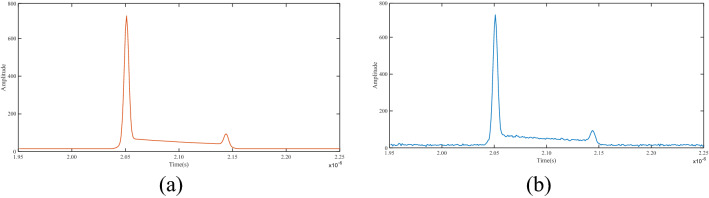
ALB simulation waveform. (**a**) Noise-free simulation waveform, (**b**) Simulation waveform with noise. ${E}_{0}=20 \mathrm{mJ}$, ${T}_{0}=5 \mathrm{ns}$, ${i}_{\mathrm{laser}}=0.3$, ${T}_{\mathrm{atm}}^{2}=0.9$, ${A}_{R}=0.025{ \mathrm{m}}^{2}$, ${\eta }_{e}=0.9$, ${\eta }_{R}=0.5$, ${k}_{d}=0.1$, ${k}_{s}=0.9$, ${F}_{r}=0.2$, *Z* = 10 m, $\beta \left(\varnothing \right)=0.0014$.

We use the above Wa-LiD model to simulate two types of echo waveform data with noise (Fig. 1b) and without noise (Fig. 1a).

#### Acquisition dataset

The measured data are acquired by the airborne laser ALB system (Mapper5000), developed by the Shanghai Institute of Optics and Fine Mechanics, the Chinese Academy of Sciences on Yuanzhi Island. Yuanzhi Island, with an area of approximately 0.3 km^2^, is a typical coral islet located in the Xisha Archipelago. The Mapper5000 system uses a four-channel high-speed waveform acquisition card for wavelengths of 1550, 1064, and 532 nm for echo collection. The sampling rate of each channel reaches 1 GSa/s, and the digital resolution is 10 bits. It includes three green channels [532 nm, photomultiplier tube (PMT)] and one near-infrared channel [1064 nm, avalanche photodiode (APD)]. The three green channels are the shallow horizontal row polarization channel (PMT1), the shallow-water orthogonal polarization channel (PMT2), and the deep-water channel (PMT3). We conducted a comparative analysis of the measured data from PMT1 and PMT2 and used the seabed echo signal extracted from the PMT3 data and the water-surface reflection signal received by the APD as the reference true value of the actual measurement for evaluating the performance of the algorithm for data processing.

### Methods

In the processing of airborne laser waveform data, suppressing the noise of the waveform data is an important step in preprocessing the waveform. The basic idea of the convolution algorithm is to regard the received waveform as the convolution of the laser emission pulse $w\left({t}_{s}\right)$ and the target cross-section $\mathrm{C}(\mathrm{t})$ with noise. Then, the received waveform can be expressed as

12 $${p}_{r}(\mathrm{t})={w}_{t}\left(t\right) \otimes \mathrm{C}\left(\mathrm{t}\right)+\mathrm{n}(\mathrm{t}) ,$$

where $\mathrm{n}(\mathrm{t})$ is the noise on the received waveform. If the influence of the noise on the received waveform is reduced as much as possible before the waveform is deconvolved, the accuracy of the waveform after deconvolution can be significantly improved. This is because when $\mathrm{n}(\mathrm{t})$ is small enough, the received waveform can be regarded simply as a laser emission pulse convolution of $w\left({t}_{s}\right)$ with the target cross-section $\mathrm{C}(\mathrm{t})$. The waveform after deconvolution is closer to the target cross-section, such that the seabed terrain will be more accurately extracted. Numerous approaches exist to denoise the waveform, for example, using filters to remove high-frequency noise or setting thresholds for the half width and amplitude of the received waveform^25^. However, the soft-threshold wavelet method produces better denoising of the deconvoluted waveform. Because the wavelet transform is a linear transform, the echo signal after orthogonal transform removes the correlation between the original signals to the maximum extent. The information from each component signal is retained, which provides a basis for the subsequent deconvolution to separate the cross-section.

#### Workflow

The experiment in this study consists of three steps. In the first step, the simulation results are used for the denoising experiment, and the simulation parameters are adjusted to maximize the approximation of the real received echo. The three main parameters in wavelet denoising (wavelet base, decomposition level, and denoising threshold) are compared and analyzed to obtain the optimal denoising parameters and lay the foundation for the following deconvolution operation.

**Figure 2:**
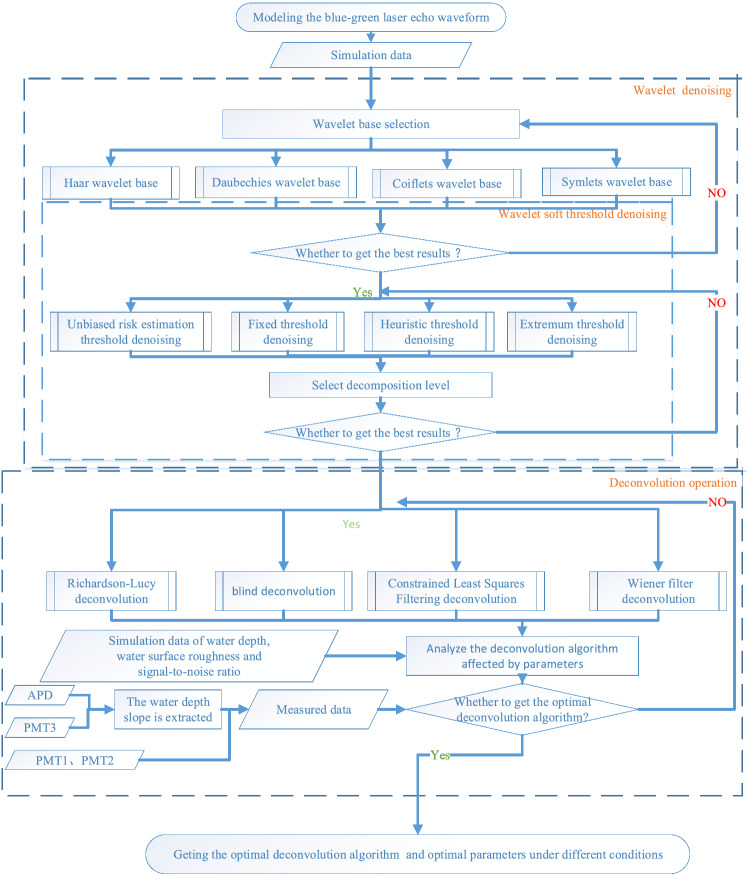
Flow chart of experimental process.

In the second step, the denoising data undergo four deconvolution operations, and the comparison with the direct deconvolution data demonstrates the necessity of denoising experiments. Simulation results are used to explore how the parameters affect the deconvolution algorithm. In the third step, the PMI1 and PMI2 channel data are used to evaluate the algorithm, where the surface position extracted from the APD data and the bottom position extracted from PMI3 are used as the true values of the measured data. Figure 2 shows a flow chart describing the experimental process.

#### Wavelet-transform denoising algorithm

The wavelet transform is divided into high-and low-frequency coefficients. The high-frequency coefficients reflect the noise and sudden changes in the signal, whereas the low-frequency coefficients reflect the overall trend and correlation of the signal^26,27^. Therefore, the high-frequency signal after wavelet transformation is subjected to wavelet threshold processing. The threshold processing function can be divided into hard and soft-threshold processing methods. The latter can be expressed as

13 $${w}_{\lambda }=\left\{\begin{array}{c}[sign(w)](\left|\left.\mathrm{w}\right|-\uplambda \right. ) \left|\left.\mathrm{w}\right|\ge\uplambda \right.\\ 0 \left|\left.\mathrm{w}\right|<\uplambda \right.\end{array}\right.,$$

where $\mathrm{w}$ is the wavelet decomposition coefficient,$\uplambda$ is the threshold, and $\mathrm{sign}(\mathrm{w})$ is the sign of w. The soft-threshold processing serves to “shrink” the wavelet coefficients, such that the input and output curves are continuous. Numerous researchers found that denoising with the soft-threshold function yields better results than the hard threshold function^28^; hence, herein we employ the soft-threshold denoising method. The wavelet-denoising threshold is mainly divided into four parts:

1. Unbiased risk-estimation threshold
  The original signal is arranged by taking the absolute value from small to large signals, squaring each, and using the square root of element *k* as the threshold. The risk expression generated by the threshold is
  14 $$\mathrm{Risk}(k)=\left[N-2k+\sum_{i=1}^{k}f\left(i\right)+\left(N-k\right)f\left(N-k\right)\right]/N,$$
  where $N$ is the total number of data, and $k$ is element *k*. Therefore, the element with the least risk serves as the unbiased risk-estimation threshold.
2. Fixed threshold
  15 $$\uplambda =\sqrt{2\mathrm{ln}(N)}.$$
3. Heursure threshold
  16 $$\mathrm{crit}=\sqrt{\frac{1}{N}\left(\frac{\mathrm{ln}(N)}{\mathrm{ln}(2)}\right)},$$
  17 $$\mathrm{eta}=[\sum_{j=1}^{N}{\left|{S}_{j}\right|}^{2}-N]/n.$$
  When eta < crit, the fixed threshold is used; otherwise, the unbiased risk threshold is employed.
4. Extreme value threshold
  18 $$\uplambda =\left\{\begin{array}{c}0.3936+0.1829\left(\frac{\mathrm{ln}N}{\mathrm{ln}2}\right), N>32\\ 0, N<32.\end{array}\right.$$

#### Waveform deconvolution algorithm

1. Wiener filter deconvolution
  WFD assumes that the signal and noise are independent, and the Wiener filter $F(t)$ is used to reduce the gap between the actual target cross-section $\mathrm{c}(\mathrm{t})$ and the evaluated target cross-section $\overline{\mathrm{c} }(\mathrm{t})$^29^. The Wiener filter $F(t)$ can be expressed in the frequency domain as
  19 $$F\left(f\right)=\frac{{\left|{W}_{t}(f)\right|}^{2}}{{\left|{W}_{t}(f)\right|}^{2}+K},$$
  where *K* is a parameter related to noise, whose value is to be determined through multiple experiments before using Wiener filtering. The time-domain estimate of the final cross-section is
  20 $$\overline{p }(t)={FFT}^{-1}\left(\frac{{P}_{r}(f)F\left(f\right)}{{W}_{t}(f)}\right).$$
2. Constrained least-square filter deconvolution
  RFD is generally used for image restoration. WFD requires that the power spectrum of the undegraded image and noise be known. These two power spectra are usually difficult to estimate, while the RFD requires only the variance and mean of the noise^30^. These parameters can be calculated from the given received waveform, which is the advantage of constrained least-square filtering^31^. The core problem of RFD is to solve the problem of the sensitivity to noise of the degradation function. “Degenerate function” is a term used in image processing. In waveform signal processing, a degenerate function may be understood as the convolution of the transmitted signal and the target cross-section. To reduce the sensitivity to noise of the convolution, a minimum criterion function C with constraints is established,
  21 $$\mathrm{C}=\sum_{0}^{M-1}{[{\nabla }^{2}w(t)]}^{2},$$
  where ${\nabla }^{2}$ is the Laplacian operator, which is used to express the smoothness of the waveform. The constraints are
  22 $${\Vert {\mathrm{p}}_{\mathrm{r}}(t)-\mathrm{D}\overline{p }(t)\Vert }^{2}={\Vert {p}_{N}\left(\mathrm{t}\right)\Vert }^{2}.$$
3. Richardson–Lucy deconvolution^32^
  RLD uses an iterative process to deconvolute the signal, which restores the likelihood of the signal by using an expectation maximization algorithm in the time domain. Iteration *i* is expressed as
  23 $${\overline{c} }^{i+1}\left(t\right)={\overline{c} }^{i}\left(t\right)\left[{w}_{t}(t) \otimes \frac{{w}_{r}(t)}{{\overline{c} }^{i}\left(t\right) \otimes {w}_{t}(t)}\right],$$
  where ${\overline{c} }^{i+1}\left(t\right)$ (t) and ${\overline{c} }^{i}\left(t\right)$ represent iteration *i* + 1 and *i*, respectively.
4. Blind deconvolution

BD is similar to RLD, except that the received signal is iterated under the assumption that the point spread function is not known, and the point spread function (transmitted signal) and the horizontal cross-sectional waveform are estimated at the same time, such that the gap between the original and the estimated cross-sections is reduced after multiple iterations^33^. Iteration *k* − 1 yields the cross-section ${c}_{k-1}$; ${{w}_{t}}_{k}$ is obtained by using the Richardson–Lucy formula, and ${c}_{k}$ is obtained from ${{w}_{t}}_{k}$. Repeated iterations give finally $\overline{c }$ and ${\overline{w} }_{t}$. The iteration formula is as follows:

24 $${\overline{{w }_{t}}}_{i+1}^{k}\left(t\right)={{{\overline{w} }_{t}}_{i}}^{k}\left(t\right)\left[{\overline{c} }^{k-1}(t) \otimes \frac{{w}_{r}(t)}{{{\overline{w} }_{t}^{k}}_{i}\left(t\right) \otimes {\overline{c} }^{k-1}(t)}\right],$$

25 $${\overline{c} }_{i+1}^{k}\left(t\right)={{\overline{c} }_{i}}^{k}\left(t\right)\left[{{\overline{w} }_{t}}^{k-1}(t) \otimes \frac{{w}_{r}(t)}{{\overline{w} }_{t}^{k}\left(t\right) \otimes {\overline{c} }^{k}(t)}\right].$$

## Results

### Experimental comparison of wavelet-denoising parameters

The quality of the wavelet-transform denoising algorithm depends mainly on the choice of wavelet base, the decomposition level, and the threshold. These three parameters are compared and verified by simulation results to provide a good data basis for the deconvolution algorithm.

#### Experiment to compare wavelet basis

**Figure 3:**
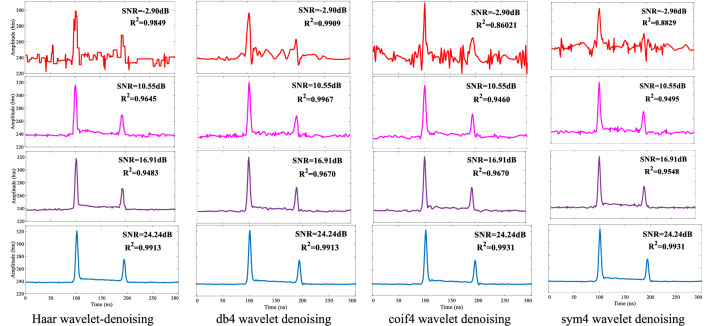
Effect of different wavelet-basis functions on waveform denoising.

Herein, we use four common wavelet bases for experiments, namely, Haar, Daubechies, Coiflets, and Symlets wavelets (abbreviated haar, db, coif, and sym wavelets). The choice of wavelet base mainly considers orthogonality, tight support, symmetry, vanishing distance, and regularity. Orthogonality indicates that the inner product of every pair of wavelet bases is zero, hence all four are orthogonal functions. Supportability means that, if the function can only take a value near zero, the range of values is called a “compact support set.” Symmetry refers to whether the wavelet-basis function is symmetric, and vanishing distance ensures that as many wavelet coefficients as possible are zero or as small as possible. The nonzero wavelet coefficients help to eliminate noise, and regularity refers to the smoothness of the wavelet-basis function. Taking a water depth of 10 m as an example, we compare four wavelet-denoising effects under different SNRs (Fig. 3). The SNR is a parameter that reflects the intensity of noise on the signal. It is expressed as

26 $$\mathrm{SNR}=10\mathrm{log}\left(\frac{{P}_{s}}{{P}_{v}}\right),$$

where ${P}_{s}$ is the average power of the noise-free signal, and ${P}_{v}$ is the average power of the noise.

To compare the denoising effects of the four wavelet bases, this study employs the goodness of fit to reflect the degree of similarity between the denoising and the original signal. The goodness of fit can be expressed as:

27 $${R}^{2}=1-\frac{\sum_{i}^{N}{\left(f\left({t}_{i}\right)-{y}_{i}\right)}^{2}}{\sum_{i}^{N}{\left(f\left({t}_{i}\right)-\overline{y }\right)}^{2}}.$$

where *N* is the number of signal samples, $f\left({t}_{i}\right)$ is the denoising signal, and ${y}_{i}$ is the original noise-free signal.

**Figure 4:**
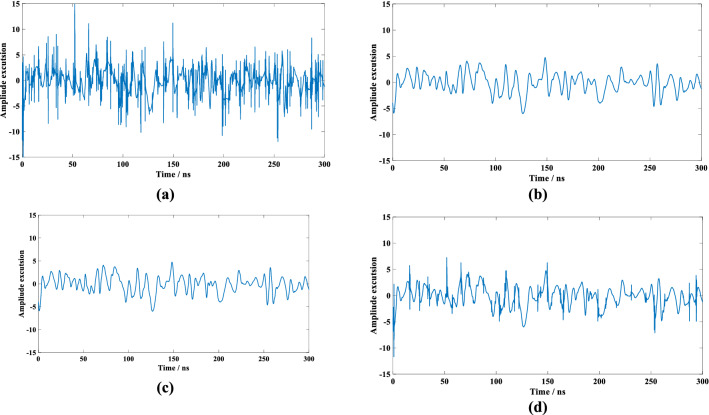
Denoising deviation diagram of various thresholds: (**a**) unbiased risk-estimation threshold denoising, (**b**) fixed threshold denoising, (**c**) heuristic threshold denoising, (**d**) extreme value threshold denoising.

Table 1 shows that the Haar wavelet is more symmetric than other wavelet-basis functions and has a smaller range of tightly supported sets. However, it is less regular than the other three wavelet bases. In the experiment, we used the fourth-order Daubechie wavelet base (db4) and the fourth-order Coiflets wavelet base (coif4). Figure 3 shows that, in the case of a low SNR and after Haar wavelet denoising, the waveform is rectangular, and other wavelet-basis denoising is relatively smooth. However, judging from the denoising of the four basic functions in Fig. 4, all four show better denoising effects when the signal-to-noise ratio is high. With decreasing SNR, the db4 and Haar wavelet bases produce better denoising than the coif and sym4 wavelet bases. For the denoising effect of the red waveform data in the fourth row of Fig. 3, the SNR is 2.90 dB, and the data after denoising by the db4 and Haar wavelets can still identify clearer reflections from the water surface and from the bottom. However, considering that the Haar wavelet is a rectangular wave, and the original waveform data are Gaussian-like waveforms, to avoid introducing further errors and to accurately extract the reflection position of the bottom echo, herein we employ the db4 wavelet basis to denoise the ALB laser echo.

**Table 1:** Wavelet-basis function performance.

Wavelet function	Haar	Daubechies	Coiflets	Symlets
Abbreviation	haar	db	coif	sym
Orthogonality	Yes	Yes	Yes	Yes
Compacted support	Yes	Yes	Yes	Yes
Symmetry	Yes	Approximate symmetry	Approximate symmetry	Approximate symmetry
Order of vanishing moments	1	N	2 N	N
Regularity	Poor	Good	Good	Good

#### Selection of wavelet-denoising threshold and decomposition layer number

This section uses the db4 wavelet-basis function to evaluate the denoising effect of the four soft thresholds. The data are acquired from a water depth of 10 m as an example, and SNR = 16.91 dB.

To clarify the comparison after denoising, Fig. 4 shows the difference between the four denoising methods and the original noise-free signal after six-layer wavelet decomposition and denoising. The unbiased risk-estimation threshold denoising performs the worst, followed by the extreme threshold denoising; the best denoising is provided by heuristic threshold denoising and fixed threshold denoising, which provide equivalent denoising.

The wavelet decomposition level is related to the data capacity and is one of the main parameters that affect denoising. Therefore, we obtain the optimal number of denoising decomposition layers of ALB by increasing the number of decomposition layers one by one. The denoising accuracy is quantified by the root mean square error (RMSE):

28 $$\mathrm{RMSE}=\sqrt{\frac{1}{N}\sum_{i=1}^{N}{(f\left({t}_{i}\right)-{y}_{i})}^{2}},$$

where *N* is the number of signal samples,$f\left({t}_{i}\right)$ is the denoising signal, and ${y}_{i}$ is the original noise-free signal.

**Figure 5:**
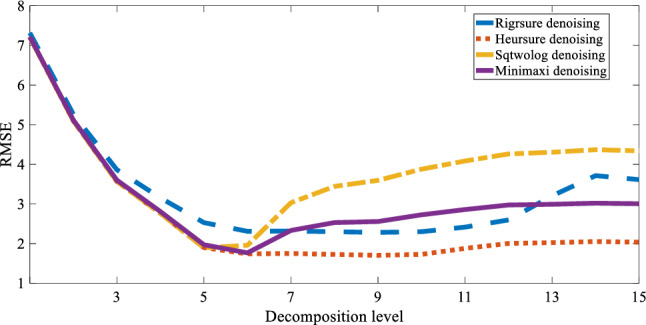
RMSE of denoising accuracy as a function of decomposition level to compare accuracies of wavelet threshold denoising decomposition levels.

Figure 5 shows the RMSE for the first to fifteenth order decomposition levels of the four threshold wavelet-denoising algorithms. These results show that the accuracy of the four soft-threshold wavelet-denoising methods has improved rapidly in the first six-order decomposition. Heuristic threshold denoising provides the highest accuracy (i.e., lowest RMSE) in the sixth order, which tends to remain stable at higher orders. At levels 7–15, the algorithms of other threshold denoising have poorer accuracy than the heuristic threshold accuracy, and the accuracy of the three threshold denoising methods that are rigrsure denoising, sqtwolog denoising and minimaxi denoising, are reduced after the six-layer decomposition. To summarize, for soft-threshold wavelet denoising, heuristic threshold denoising provides the best echo signal denoising, and six decomposition layers are optimal for denoising.

### Experiment to compare deconvolution algorithms

To explore how the wavelet-denoising algorithm affects the deconvolution algorithm, Fig. 7 shows how the deconvolution operation affects the data before and after wavelet denoising and compares the effect of data deconvolution.

**Figure 6:**
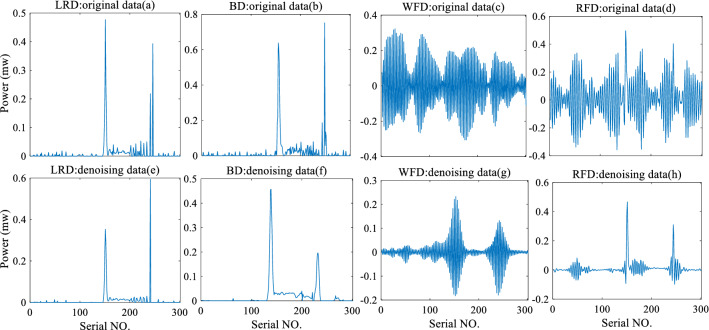
Comparison of deconvolution algorithms. The first row (**a**), (**b**), (**c**), and (**d**) show the deconvolution effect without wavelet denoising. The second row (**e**), (**f**), (**g**), and (**h**), is the effect picture of deconvolution operation after wavelet denoising.

Figure 6 shows that the data after wavelet denoising yields a better deconvolution. Figure 6a,b,e,f show that the waveform of the denoising data becomes smooth after deconvolution, and the reflected echo is retained. Because the deconvolution operation can improve the data resolution, it also amplifies the impact of noise on the data. In Fig. 6a, significant noise interference appears around the bottom reflection echo. However, wavelet denoising removes the interference, thereby improving the strength of the bottom reflection. The effect of wavelet denoising is clearer with the WFD and RFD algorithms. Before wavelet denoising, the noise of the WFD and RFD algorithm solution results is completely fused with the target reflection signal, and effective information cannot be extracted. After wavelet denoising, the noise is suppressed, and the target reflection becomes more evident.

## Discussion

### Comparative analysis of simulation data

To further analyze how processing affects each deconvolution algorithm, we add the following algorithm-evaluation indicators to evaluate how processing affects the deconvolution algorithm:

1. Root mean square error of water-depth slope distance
  Because the deconvolution algorithm restores the cross-sectional shape of the target, evaluating the accuracy of the water-depth data better reflects the quality of the algorithm. The *RMSE* of the water-depth slope distance is expressed as:
  29 $${RMSE}_{D}=\sqrt{\frac{\sum_{i}^{N}{({Z}_{i}^{^{\prime}}-{Z}_{i})}^{2}}{N}},$$
  where ${Z}_{i}^{\mathrm{^{\prime}}}$ and ${Z}_{i}$ are the estimated aquatic slope distance and the true water depth slope distance, respectively, and $N$ is the number of samples.
2. Correlation coefficient
  We calculate the correlation coefficient (CORR) between the deconvolution result and the true reflected echo:
  30 $$CORR=\frac{\sum_{i}^{N}({M}_{i}^{^{\prime}}-{\overline{M} }_{i}^{^{\prime}})({M}_{i}-\overline{M })}{\sqrt{\sum_{i}^{N}{({M}_{i}^{^{\prime}}-{\overline{M} }_{i}^{^{\prime}})}^{2}\sum_{i}^{N}{({M}_{i}-\overline{M })}^{2}}},$$
  where ${M}_{i}^{\mathrm{^{\prime}}}$ is the waveform processed by the algorithm, and ${M}_{i}$ is the reflection from the bottom. The closer the correlation coefficient is to unity, the stronger the correlation is between the two.
3. Water-depth goodness of fit
  31 $${{R}_{z}}^{2}=1-\frac{\sum_{i}^{N}{\left({Z}_{i}^{^{\prime}}-{Z}_{i}\right)}^{2}}{\sum_{i}^{N}{\left({Z}_{i}-\overline{Z }\right)}^{2}}.$$
  The water depth goodness of fit ${{R}_{z}}^{2}$ describes the degree of fit between the extracted water depth and the actual water depth. The fitting improves as ${{R}_{z}}^{2}$ approaches unity.
4. The time *T* required to evaluate the computational efficiency of the algorithm.

Table 2 lists the results of the deconvolution of 100 sets of wavelet denoising and denoising simulation data. The RFD algorithm is the most accurate and requires the shortest calculation time. The accuracy of the WFD algorithm is relatively poor, but over the course of the experiment, the RFD algorithm must repeatedly adjust the convolution parameters to achieve the optimal effect. The RLD algorithm deconvolutes the waveform through iteration, which takes a relatively long time. However, unlike the RLD algorithm, the BD algorithm assumes that the point spread function is not known; hence it must be estimated before running the BD algorithm. This increases the calculation time and reduces the accuracy.

**Table 2:** Comparison of performance of deconvolution algorithms.

Evaluation parameter	RLD	BD	WFD	RFD
RMSE (m)	0.1015	0.4220	0.6059	0.0435
CORR	0.8226	0.8791	0.8262	0.8516
R_z_^2^	0.9910	0.8837	0.9663	0.9664
T (s)	0.8194	0.9249	0.3681	0.4345

**Figure 7:**
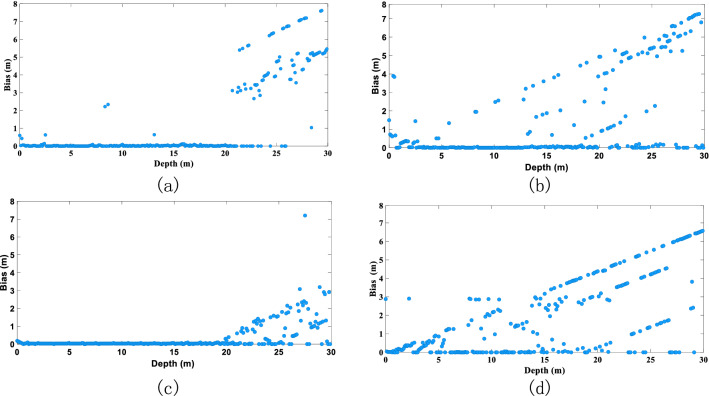
Influence of water depth on accuracy of water-depth extraction: (**a**) RLD processing, (**b**) BD processing, (**c**) RFD processing results, and (**d**) WFD processing results (the graphs show the absolute value of the bias).

**Figure 8:**
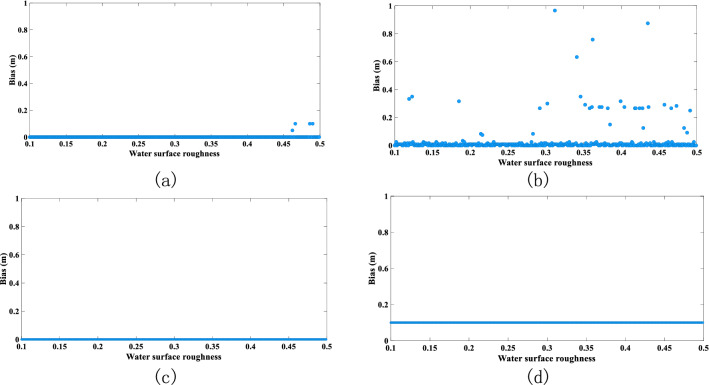
Effect of water-surface roughness on accuracy of water-depth extraction. (**a**) RLD processing, (**b**) BD processing, (**c**) RFD processing, and (**d**) WFD processing (graphs show the absolute value of the bias).

**Figure 9:**
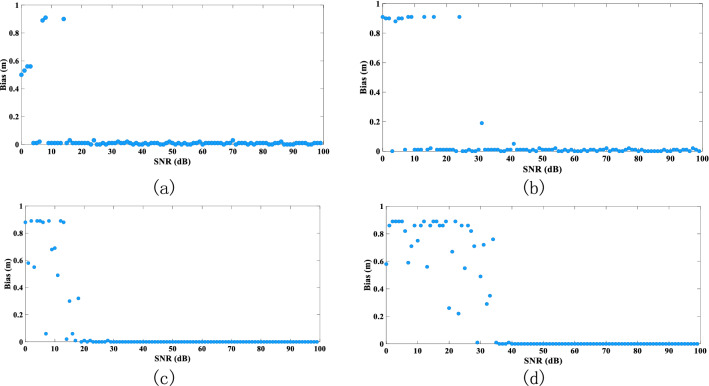
Influence of SNR on accuracy of water-depth extraction: (**a**) RLD processing, (**b**) BD processing, (**c**) RFD processing, and (**d**) WFD processing (graphs show the absolute value of the bias).

Based on the deconvolution performance of each algorithm, we further explore how changes in the main parameters of the laser echo affect the algorithms (see Figs. 7, 8, 9).

Water depth is one of the main factors that affect the accuracy of extraction of the water-depth slope distance. If the water is too shallow, the surface reflection overlaps with the bottom reflection, and the water depth cannot be extracted. If the water is too deep, the bottom reflection is masked by noise, making it impossible to extract an effective reflection signal. This is apparent from the deviation values extracted from the four groups in Fig. 7: The extraction of the water-depth slope distance is more accurate for water depths of 3–20 m, after which the accuracy begins to decrease. For depths greater than 25 m, the accuracy decreases significantly. Of the four deconvolution algorithms, the RFD algorithm is the most stable against water-depth interference, whereas the WFD performance is relatively unstable (WFD is most affected for the water depths of 0–5 m).

Figure 8 shows that the BD algorithm performs poorly when considering water-surface roughness. However, compared with the influence of the other parameters, the water-surface roughness produces only a small effect on the four deconvolution algorithms. This result is attributed to the fact that the rough water surface primarily affects the water-surface reflection, which is the first reflection received by the receiver, and it only transits through air, resulting in a strong signal. Consequently, this signal is only slightly affected by water depth. However, in the process of converting the water-depth slope distance to the actual water depth, the water-surface roughness becomes one of the main factors affecting the actual water-depth conversion, because a rougher water surface leads to a greater difference between the slope distance and the actual water depth.

Noise is also an important factor affecting water-depth extraction. When the SNR < 20 dB, the extraction accuracy of the water-depth slope varies differently for the four deconvolution algorithms (WFD is the least stable, and RLD is the most stable). When SNR > 40 dB, the four algorithms perform well. Performing wavelet denoising before convolution thus improves the SNR and the accuracy of water-depth extraction.

Comparing the four deconvolution algorithms shows that RLD and BD are affected similarly by convolution, and the same is true of WFD and RFD. The RLD and RFD algorithms are more robust, whereas the WFD algorithm is not. Although RFD is affected by intermittent pulses, it experiences a slight peak broadening, which is conducive to the extraction of peak time intervals. The RFD algorithm is relatively stable.

### Comparative analysis of measured data

Before processing, the PMI1 and PMI2 channel data, the infrared channel, and the deep-water channel data are preprocessed: The reflection from the water surface is extracted from the APD data, and the bottom reflection is extracted from the PMI3 deep-water channel data. These data are used as the true values for the measured data. On the one hand, a comparison of the measured data explores the performance and difference of the deconvolution algorithm in the positive channel and the orthogonal channel. On the other hand, it tests the effectiveness of the algorithm proposed herein, and the deconvolution algorithms are compared with the traditional peak detection method (PD), which is to extract the peaks directly from the original data, such that the PD algorithm does not need to calculate the two parameters of goodness of fit and correlation coefficient. Based on the characteristics of the laser reflection waveform and the simulation results, we divide the reflection data into three groups of water-depth slopes: 0–5 , 5–25, and > 25 m. Each group of data has 5000 original reflection signals. The experiments were conducted on the PMT1 and PMT2 channel data (see Tables 3, 4, 5, 6, 7, 8).

**Table 3:** Algorithm extraction compared with measured data (water-depth slope distance is 0–5 m, PMT1).

Evaluation parameter	PD	RLD	BD	WFD	RFD
RMSE (m)	0.5299	0.1537	0.1545	0.3841	0.1945
R_z_^2^	–	0.9405	0.9435	0.9924	0.9318
CORR	–	0.8279	0.8293	0.0789	0.7246
T (s)	0.1363	14.7515	35.8408	11.4099	14.8150

**Table 4:** Algorithm extraction compared with measured data (water-depth slope distance is 0–5 m, PMT2).

Evaluation parameter	PD	RLD	BD	WFD	RFD
RMSE (m)	0.5462	0.1537	0.1545	0.2089	0.1945
R_z_^2^	–	0.9405	0.9435	0.9143	0.9318
CORR	–	0.8279	0.8293	0.9079	0.7246
T (s)	0.1155	14.7713	35.4752	14.6952	14.7664

**Table 5:** Algorithm extraction compared with measured data (water-depth slope distance is 5–25 m, PMT1).

Evaluation parameter	PD	RLD	BD	WFD	RFD
RMSE (m)	0.4445	0.0293	0.0289	0.2437	0.0420
R_z_^2^	–	0.9975	0.9985	0.7806	0.9786
CORR	–	0.9225	0.9237	0.0698	0.3206
T (s)	0.1016	16.1511	48.9224	12.7001	15.1381

**Table 6:** Algorithm extraction compared with measured data (water-depth slope distance is 5–25 m, PMT2).

Evaluation parameter	PD	RLD	BD	WFD	RFD
RMSE (m)	0.4923	0.0279	0.0279	0.2315	0.0282
R_z_^2^	–	0.9968	0.9970	0.8258	0.9999
CORR	–	0.9150	0.9151	0.0805	0.8057
T (s)	0.1028	17.6797	48.4024	12.3284	16.7678

**Table 7:** Algorithm extraction compared with measured data (WFD did not detect weak bottom reflection; water-depth slope distance is > 25 m, PMT1).

Evaluation parameter	PD	RLD	BD	WFD	RFD
RMSE (m)	1.3606	0.2547	0.2561	–	0.4530
R_z_^2^	–	0.7326	0.7345	–	0.8244
CORR	–	0.9221	0.9229	–	0.7904
T (s)	0.1381	19.1912	50.5954	–	17.0388

**Table 8:** Algorithm extraction compared with measured data (WFD did not detect weak bottom reflection; water-depth slope distance is > 25 m, PMT2).

Evaluation parameter	PD	RLD	BD	WFD	RFD
RMSE (m)	1.5700	0.2624	0.2666	–	0.3935
R_z_^2^	–	0.6678	0.6672	–	0.8880
CORR	–	0.8960	0.8957	–	0.7598
T (s)	0.1111	18.6591	50.5167	–	17.1501

Tables 3 and 4 show the accuracy of shallow-water waveform extraction using the extraction results of the four algorithms: The RLD and BD algorithms are more accurate than the WFD and RFD algorithms. Because the BD algorithm is a deconvolution operation for which the original function is assumed to be unknown, the calculation time is significantly longer than that of the other algorithms, and the accuracy of the BD algorithm approaches that of the RLD algorithm, Although the PD algorithm has a shorter running time, its accuracy is significantly lower than that of the deconvolution algorithms. The comparison of extracting via PMT1 channel data versus PMT2 channel data shows that the latter is more accurate than the former, and the accuracy of the WFD algorithm applied to PMT2 is significantly improved. A comprehensive analysis shows that the RLD algorithm works best with PMT2 channel data for extracting shallow-water depth.

Tables 5 and 6 show the extraction accuracy of medium water depth, which is significantly improved compared to the accuracy of the shallow-water experiment. Of the four deconvolution algorithms, the BD algorithm is the most accurate, but has the longest calculation time. The accuracies of the RLD and RFD algorithms are similar to each other, and the correlation coefficient of RLD is better than that of RFD, but the computational efficiency of RFD is better than that of RLD. Both RLD and RFD algorithms perform well for extracting medium water depth. The accuracy of the PD algorithm is slightly improved, but the accuracy is still far lower than that of the deconvolution algorithms. Comparing the precision of the extraction via PMT1 with that via PMT2 shows that PMT2 channel data are more conducive to deconvolution processing, which improves the accuracy of all four algorithms.

Tables 7 and 8 do not evaluate the extraction parameters of the WFD algorithm, because the bottom reflection is weak. After deconvolution, numerous waveforms involve no reflection from the bottom, such that the algorithm’s performance in deep waters is unavailable. In deep waters, the RLD algorithm provides the most accurate deconvolution extraction, although the accuracy of the BD algorithm approaches that of the RLD algorithm; however, the accuracy of the PD algorithm decreases most rapidly. For water depth > 25 m, the RLD and BD algorithms provide better results with PMT1 data, whereas the RFD algorithm provides better results with PMT2 data.

These results are apparent upon comparing the three groups of extraction parameters. The slope distance is most accurate for the extracted water depth of 5–25 m. The extraction accuracy decreases for a water-depth slope distance less than 5 m or greater than 25 m. The measured data are consistent with the simulated results, which truly reflect how the parameters affect the algorithm. Although the PD algorithm has a shorter operation time, its accuracy is exacerbated compared to that of the deconvolution algorithms, and it is considerably affected by the water depth. The deconvolution algorithm based on wavelet denoising is more conducive to the water-depth extraction of the ALB system.

Among the four deconvolution algorithms, although the WFD algorithm provides poor extraction accuracy for water depths greater than 25 m, the other three algorithms all show provide good accuracy and robustness. The BD algorithm requires a long computation time, making it rather inefficient. The RLD algorithm processes PMT1 (PMT2) data for water depths less than (greater than) 25 m. However, because the RLD algorithm is iterative, the RFD algorithm is more efficient. Further, the RFD algorithm applied to the PMT2 data leads to more accurate results than when applied to the PMT1 data, such that either the RLD or the RFD algorithm must be used, depending on the actual situation.

## Conclusion

This study compares the simulated results with measured data for wavelet transforms, soft-threshold selection, and deconvolution. The main conclusions may be summarized as follows:

1. For selecting wavelet bases, both the db4 wavelet and Haar wavelet bases offer good noise suppression. The Haar wavelets form a rectangular wave, and that the laser emission waveform is Gaussian. To avoid introducing more errors and to accurately extract the position of the bottom reflection, the db4 wavelet base must be used to denoise the ALB laser echo.
2. To select a soft threshold, the heuristic threshold denoising method is equivalent to the fixed threshold denoising method, and both are better than the extreme threshold denoising method. Based on layer-by-layer decomposition, the heuristic threshold is best for echo signal denoising, and six decomposition layers are optimal for denoising.
3. A comparative analysis of data deconvolution before and after wavelet denoising shows that, after wavelet denoising, the WFD and RFD algorithms perform significantly better for extracting reflections from the target, and the performance of RLD and BD algorithms likewise improves after wavelet denoising. The deconvolution experiment with the SNR model shows that a high SNR guarantees effective deconvolution. Therefore, performing a wavelet transform before the deconvolution experiment improves the deconvolution, which is particularly helpful for the RFD algorithm.
4. The echo parameter has a varying effect on the algorithm: The RLD and RFD algorithms are more robust against such variations, whereas the WFD algorithm is less robust. Although RFD produces intermittent pulses, it has only small peak broadening, which facilitates the extraction of peak time intervals. Furthermore, the algorithm is stable.
5. For multichannel data in actual experiments, both the RLD and the RFD algorithms are effective and robust. Although the accuracy of the BD algorithm is similar to that of the RLD algorithm (or even superior to a certain extent), it requires a longer calculation time and thus has low computational efficiency, making it unsuitable for large-scale data calculation. The RFD algorithm is more suited for PMT2 data than for PMT1 data. Compared with the RLD algorithm, RFD avoids the iterative process, and the solution accuracy is similar to that of RLD. Thus, RFD provides a new opportunities for ALB deconvolution algorithms.
